# The effect of adenotonsillar hypertrophy and other obstructive diseases on sleep disorders in children^☆^

**DOI:** 10.1016/j.bjorl.2018.06.004

**Published:** 2018-08-07

**Authors:** Fatma Caylakli

**Affiliations:** Baskent University School of Medicine, Otorhinolaryngology Head and Neck Surgery Department, Ankara, Turkey

Dear Editor,

I read the manuscript entitled “*Sleep disorders in children with moderate to severe persistent allergic rhinitis*” by Loekmanwidjaja, et al.^1^ The manuscript is so valuable, it is about the evaluation of the interference of allergic rhinitis on sleep disorders in children. In sleep disorders, diseases causing nasal congestion and upper airway obstruction are always important. Besides allergic rhinitis, infectious diseases and hypertrophy of adenoid and tonsillar tissues are also conditions that affect sleep disorders.^2^ Allergic rhinitis has also effect on adenotonsillar hypertrophy.^3^, ^4^ Tonsillar hypertrophy has a great role in obstructing upper airway, and consequently in sleep disorders.^5^ This manuscript discussed allergic rhinitis in children with sleep disorders, but we cannot omit adenotonsillar hypertrophy and other obstructive conditions. When speaking about sleep disorders in children, it would be better to bring also up some discussion regarding adenotonsillar hypertrophy and other obstructive diseases, not only allergic rhinitis.

## Conflicts of interest

The author declares no conflicts of interest.
